# Inferior Vena Cava Cement Migration with Pulmonary Cement Embolism: A Case Report

**DOI:** 10.5811/cpcem.64561

**Published:** 2026-09-08

**Authors:** Christina Sneed, Syed Adnan

**Affiliations:** Jefferson Einstein Montgomery Hospital, Department of Emergency Medicine, Norristown, Pennsylvania

**Keywords:** pulmonary embolism, vertebral augmentation, kyphoplasty, cement

## Abstract

**Introduction:**

Vertebral augmentation procedures, including vertebroplasty and kyphoplasty, can result in cement extravasation, which is often clinically silent. Less commonly, cement may migrate intravascularly into the inferior vena cava (IVC) and pulmonary arterial circulation, resulting in pulmonary cement embolism, a potentially life-threatening complication. Early recognition in the emergency department (ED) is essential to guide appropriate imaging, specialty consultation, and timely management.

**Case Report:**

A 76-year-old woman with recent third lumbar vertebral augmentation presented to the ED with acute worsening low back pain and burning bilateral radicular symptoms approximately one week after the procedure. Computed tomography (CT) of the abdomen and pelvis with intravenous contrast demonstrated linear hyperdense material extending from the treated vertebral body into the IVC, concerning for intravascular cement migration. Additional CT of the chest demonstrated multiple bilateral subsegmental pulmonary cement emboli. Interventional radiology was consulted emergently, and the patient was transferred for endovascular retrieval of the IVC cement fragment, which was successfully removed. The pulmonary cement emboli were small, peripheral, and asymptomatic and were managed conservatively. The patient remained hemodynamically stable and was discharged after monitored hospitalization.

**Conclusion:**

Inferior vena cava cement migration with pulmonary cement embolism is a potentially life-threatening complication of vertebral augmentation. Emergency physicians should maintain suspicion for this diagnosis in patients with acute pain or new cardiopulmonary symptoms following recent spinal procedures. Early imaging, multidisciplinary consultation, monitored admission, and endovascular retrieval when indicated may reduce morbidity.

## INTRODUCTION

Vertebral augmentation procedures, including vertebroplasty and kyphoplasty, are widely performed for osteoporotic compression fractures. Cement leakage is common, with reported rates of 30–70%, although most cases are asymptomatic and clinically insignificant.^1^,^2^ Venous extravasation through the vertebral venous plexus can result in migration into segmental veins, the inferior vena cava (IVC), and pulmonary arterial circulation.^3^ Pulmonary cement embolism is the most recognized serious vascular complication, with reported incidence of 3–23%.^4^ While many emboli are clinically silent, larger fragments can cause hypoxia, right ventricular strain, or cardiovascular collapse.^3^,^5^ Although pulmonary cement embolism has been reported after vertebral augmentation, cement migration into the IVC is less frequently described and appears to be limited to isolated case reports and small series, yet it may pose substantial risk of distal embolization requiring urgent intervention.^6^,^9^

## CASE REPORT

A 76-year-old woman with a history of transcatheter aortic valve replacement, atrial fibrillation, congestive heart failure, and recent third lumbar (L3) vertebral compression fracture status post vertebral augmentation approximately one week prior presented to the emergency department (ED) with acute worsening low back pain and burning bilateral radicular symptoms.

The vertebral augmentation had been performed in an outpatient setting, and procedural records and current medication records were unavailable at the time of presentation. On arrival, vital signs were as follows: temperature, 36.8 °C; heart rate, 117 beats per minute; respiratory rate, 18 breaths per minute; blood pressure, 126/66 mm Hg; and oxygen saturation, 95–99% on room air. Her vital signs remained stable during the ED course prior to transfer, with heart rate ranging from the 90s to a maximum of 120 beats per minute.

The patient was awake, alert, appropriately conversational, nontoxic appearing, and in no acute cardiopulmonary distress. Pulmonary examination demonstrated equal bilateral breath sounds without rales or rhonchi. Cardiovascular examination demonstrated tachycardia with regular rhythm. Physical examination revealed extensive ecchymoses over the abdomen and posterior trunk, midline tenderness of the mid-to-lower lumbar spine, and gluteal tenderness over the hematoma site. Neurologic examination demonstrated intact sensation and 5/5 motor strength in the bilateral upper and lower extremities without saddle anesthesia. Gait assessment was deferred because of pain.

Laboratory evaluation demonstrated hemoglobin of 8.3 g/dL (reference range, 12.0–16.0 g/dL) without prior values available for comparison, limiting determination of chronicity. High-sensitivity troponin levels were minimally elevated but stable on serial testing at 22, 18, and 18 ng/L (≤13 ng/L), and brain natriuretic peptide was elevated at 326 pg/mL (0.0–100.0 pg/mL), concerning for possible right heart strain. Electrocardiogram demonstrated sinus tachycardia without ischemic changes, and the stable serial cardiac biomarkers were not suggestive of acute myocardial ischemia. There were no electrolyte derangements, acute kidney injury, or leukocytosis suggestive of infectious pathology.

Computed tomography (CT) of the abdomen and pelvis with intravenous contrast demonstrated postsurgical changes consistent with prior vertebral cement augmentation at L3 and linear hyperdense material extending from the treated vertebral body into the IVC, concerning for intravascular cement migration (Image 1). The study also demonstrated a 5.7 × 12.1 cm hematoma in the left superior gluteal region with surrounding subcutaneous edema.

Given concern for distal embolization, interventional radiology was consulted emergently. After discussion with the consulting team, CT of the chest without contrast demonstrated multiple branching high-attenuation foci within bilateral subsegmental pulmonary arteries, consistent with pulmonary cement emboli (Image 2). Additional findings included an age-indeterminate 12^th^. thoracic vertebral compression deformity without bony retropulsion and incidental pulmonary nodules. The patient was transferred to a tertiary care center where interventional radiology successfully performed endovascular retrieval of the IVC cement fragment without complication. The pulmonary cement emboli were small, subsegmental, and asymptomatic and were managed conservatively.

*CPC-EM Capsule*

What do we already know about this clinical entity?

*Pulmonary cement embolism is an uncommon but recognized complication of vertebral augmentation procedures.*

What makes this presentation of disease reportable?

*Computed tomography showed cement migration from a lumbar vertebral augmentation site into the inferior vena cava and bilateral pulmonary arteries.*

What is the major learning point?

*Prior vertebral augmentation should prompt consideration of cement embolism, especially when characteristic hyperdense intravascular findings are present.*

How might this improve emergency medicine practice?

*Awareness of this rare complication may prevent missed diagnoses and facilitate timely consultation and management.*

Postprocedure, the patient remained hemodynamically stable. Anticoagulation was initially held because of the large gluteal hematoma and extensive ecchymoses. Transthoracic echocardiogram demonstrated a left ventricular ejection fraction of 75%, a dilated right ventricle with moderately reduced right ventricular function, severe mitral stenosis, and a normally functioning transcatheter aortic valve replacement. Cardiology recommended outpatient follow-up for mitral stenosis management. Given her history of atrial fibrillation, warfarin with enoxaparin bridging was later initiated, targeting an international normalized ratio of 2–3. The patient was monitored on telemetry during hospitalization and discharged on hospital day 10. She was subsequently lost to follow-up.

## DISCUSSION

Cement leakage is a frequent radiographic finding after vertebral augmentation, occurring in 30–70% of procedures, although intravascular migration is less common.^1^,^2^ Fragments can travel through the vertebral venous plexus into the IVC, with distal pulmonary embolization reported after vertebral augmentation in the literature.^3^–^5^,^7^ Pulmonary cement embolism is the most frequently reported serious vascular complication of vertebral augmentation.^3^–^5^ Although many pulmonary cement emboli are asymptomatic, emboli that are larger, central, or multiple can be life-threatening, particularly in patients with pre-existing cardiopulmonary disease.^3^,^5^,^8^ Mechanistically, embolized polymethylmethacrylate can cause mechanical obstruction of the pulmonary arterial vasculature, increasing pulmonary vascular resistance and precipitating right ventricular strain or hemodynamic compromise. In addition, rigid cement fragments may continue to migrate centrally into the right heart, where they can cause arrhythmia, valvular injury, cardiac perforation, hemopericardium, tamponade, or death. Some reports also suggest endothelial injury and secondary thrombosis may further contribute to cardiopulmonary sequelae.^5^,^6^,^8^,^9^

In this case, the patient’s history of transcatheter aortic valve replacement, atrial fibrillation, and postprocedural echocardiographic evidence of right ventricular dilation with moderately reduced right ventricular function increased concern for possible hemodynamic compromise despite the absence of overt hypoxia or instability.

The vertebral level of augmentation may influence the risk and pattern of cement migration due to venous anatomy. The L3 vertebral body drains into the internal vertebral venous plexus, which communicates directly with the IVC, providing a relatively short route for embolization.^3^,^7^ In this patient, the L3 origin may have facilitated migration into the pulmonary arterial circulation. The combination of an IVC cement fragment and multiple pulmonary cement emboli, along with echocardiographic evidence of right ventricular dysfunction, supported prompt interventional radiology consultation, monitored admission, and urgent endovascular retrieval of the intravascular fragment.^6^,^9^

Management of pulmonary cement embolism depends on symptom burden, embolus location, and hemodynamic status. Small, peripheral, and asymptomatic emboli may be managed conservatively, whereas symptomatic, central, or hemodynamically significant emboli may require anticoagulation or urgent interventional retrieval.^5^,^8^ Endovascular retrieval of IVC cement fragments is increasingly described in the literature, and dual venous access with large-bore sheath protection may reduce the risk of distal embolization during retrieval.^6^,^9^

This case highlights several important considerations for emergency physicians. Recent vertebral augmentation should prompt consideration of intravascular cement migration in patients presenting with acute back pain, radicular symptoms, unexplained cardiopulmonary findings, or suspected pulmonary embolic phenomena, particularly because traditional thromboembolic risk stratification tools may not account for nonthrombotic complications such as pulmonary cement embolism. Early CT can identify both the source fragment and distal embolization. Prompt multidisciplinary consultation with interventional radiology and monitored admission are essential when embolization risk exists. Finally, anticoagulation decisions in pulmonary cement embolism should be individualized, particularly when concurrent bleeding risk is present.

## CONCLUSION

Inferior vena cava cement migration with pulmonary cement embolism is a rare but potentially life-threatening complication of vertebral augmentation. Emergency physicians should consider this diagnosis in patients presenting after recent spinal procedures with acute pain or unexplained cardiopulmonary symptoms. Early imaging, specialty consultation, and monitored management may reduce morbidity.

## Figures and Tables

**Image 1:**
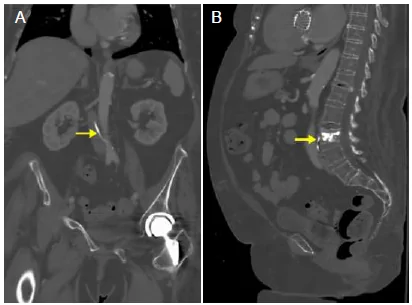
A) Coronal and B) sagittal contrast-enhanced computed tomography of the abdomen and pelvis demonstrating linear hyperdense cement extending from the third lumbar vertebral augmentation site into the inferior vena cava (arrow), concerning for intravascular cement migration.

**Image 2:**
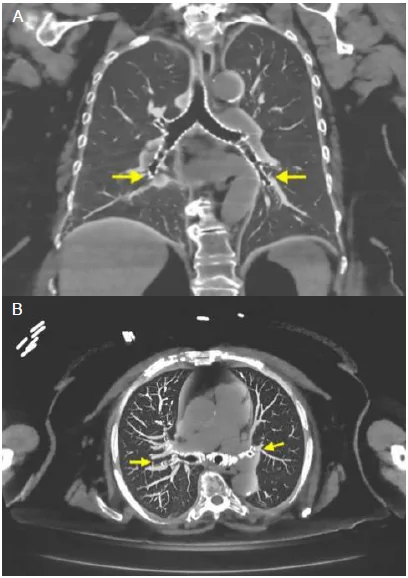
A) Coronal and B) axial contrast-enhanced computed tomography demonstrating branching hyperattenuating cement emboli within bilateral subsegmental pulmonary arteries (arrows).
